# Global report on preterm birth and stillbirth (6 of 7): ethical considerations

**DOI:** 10.1186/1471-2393-10-S1-S6

**Published:** 2010-02-23

**Authors:** Maureen Kelley, Craig E Rubens, the GAPPS Review Group

**Affiliations:** 1 Department of Pediatrics, Bioethics Division, University of Washington School of Medicine and Treuman Katz Center for Pediatric Bioethics, Seattle Children's Hospital, Seattle, Washington, USA; 2 Global Alliance to Prevent Prematurity and Stillbirth, an initiative of Seattle Children's, Seattle, Washington, USA; 3 Department of Pediatrics at University of Washington School of Medicine, Seattle, Washington, USA

## Abstract

**Introduction:**

Despite the substantial global burden of preterm and stillbirth, little attention has been given to the ethical considerations related to research and interventions in the global context. Ethical dilemmas surrounding reproductive decisions and the care of preterm newborns impact the delivery of interventions, and are not well understood in low-resource settings. Issues such as how to address the moral and cultural attitudes surrounding stillbirths, have cross-cutting implications for global visibility of the disease burden. This analysis identifies ethical issues impacting definitions, discovery, development, and delivery of effective interventions to decrease the global burden of preterm birth and stillbirth.

**Methods:**

This review is based on a comprehensive literature review; an ethical analysis of other articles within this global report; and discussions with GAPPS's Scientific Advisory Council, team of international investigators, and a community of international experts on maternal, newborn, and child health and bioethics from the 2009 International Conference on Prematurity and Stillbirth. The literature review includes articles in PubMed, Academic Search Complete (EBSCO), and Philosopher's Index with a range of 1995-2008.

**Results:**

Advancements in discovery science relating to preterm birth and stillbirth require careful consideration in the design and use of repositories containing maternal specimens and data. Equally important is the need to improve clinical translation from basic science research to delivery of interventions, and to ensure global needs inform discovery science agenda-setting. Ethical issues in the development of interventions include a need to balance immediate versus long-term impacts—such as caring for preterm newborns rather than preventing preterm births. The delivery of interventions must address: women's health disparities as determinants of preterm birth and stillbirth; improving measurements of impact on equity in coverage; balancing maternal and newborn outcomes in choosing interventions; and understanding the personal and cross-cultural experiences of preterm birth and stillbirth among women, families and communities.

**Conclusion:**

Efforts to improve visibility, funding, research and the successful delivery of interventions for preterm birth and stillbirth face a number of ethical concerns. Thoughtful input from those in health policy, bioethics and international research ethics helped shape an interdisciplinary global action agenda to prevent preterm birth and stillbirth.

## Introduction

The last decade in global health has seen one of the most exciting paradigm shifts in scientific research and investment: "Good science" now means more than rigorous application of scientific methods toward important scientific discoveries. Good science has also come to mean a deliberate attempt to direct methodologically rigorous science toward the disease burden of the underserved, across borders. With this move, the role for ethics in science is becoming more than an important constraint on scientific practice and unintended consequences of unbridled discovery. Ethics can also inform and shape the research agendas for institutions and stakeholders interested in improving lives and alleviating suffering among populations whose burden remains underrepresented on academic, political, and investment agendas.

Empirical ethics is also emerging as a respected mode of inquiry in social science. It provides a critical source of empirical data to improve the ethically responsible conduct of research, develop culturally and ethically sensitive delivery of interventions, identify ethically significant blind spots in the measurement of the disease burden, and inform policy change. It is in this spirit that this scientific report integrated ethics into the science of maternal, newborn and child health with regard to the global burden of preterm birth and stillbirth.

This article offers a systematic and detailed review of the ethical issues that informed, or were raised, during deliberations surrounding the report. It considers deeper concerns and controversies that will take time to address with interdisciplinary inquiry and deliberation. This article builds upon the existing ethics and social science literature in population health, social justice and global health, international research ethics, neonatal ethics, and health and human rights. Lastly, this article identifies issues raised specifically by the global health burden of preterm birth and stillbirth that have not been well addressed in existing literature.

Despite the significant global burden of preterm birth and stillbirth, no systematic international survey of relevant ethical and social justice issues exists. The 2007 Institute of Medicine (IOM) report,* Preterm Birth: Causes, Consequences, and Prevention,* includes in its appendices a review of the ethical issues involved in preterm birth in the United States [1]. While the IOM report is valuable for canvassing ethical concerns of preterm birth in high-income countries (HICs), significant gaps remain in understanding ethical and social justice issues surrounding preterm birth and stillbirth in low- and middle-income countries (LMICs).

The purpose of the ethics review is to facilitate dialogue with scientific investigators and to better identify areas for targeted normative and empirical bioethics research with high impact. Specifically, the analysis highlights ethical issues that directly or indirectly impact definitions, discovery science, development, and equitable delivery of effective interventions to decrease the global burden of preterm birth and stillbirth.

## Methods

This ethics review is based on a comprehensive literature review, an ethical analysis of the scientific gap analysis on preterm birth and stillbirth interventions, and discussions with GAPPS's Scientific Advisory Council and team of international investigators, and a community of international experts on maternal, newborn, and child health from the International Conference on Prematurity and Stillbirth, Seattle, WA, USA (May 2009). An Ethics and Social Justice working group convened to discuss the top ethical concerns identified in an early draft of this article through ethical cases from the field, offered by conference participants prior to the meeting. The group served an advisory role to the broader gap analysis and core investigator team. As such, many of the key concerns raised in the ethics working group are represented here. Revisions were made to reflect the working group discussions. The specific recommendations of that group will be published as a separate document to represent the full diversity of opinions and issues that could not be covered in detail in this review. Several of the research questions listed in the conclusion of this article have been taken up by the working group's members following the conference.

The literature review covered peer-reviewed articles in PubMed, Academic Search Complete (EBSCO), and Philosopher's Index with a range of 1995-2008 for the general search terms. The following six disciplines are included: (1) bioethics, (2) philosophy, (3) social sciences, (4) medicine, (5) public health, and (6) epidemiology. Commentary and empirical studies appearing in peer-reviewed medical journals are included, as are scientific studies containing a substantive discussion of ethical issues or equity concerns related to preterm birth or stillbirth. The review did not include legal or policy documents beyond U.S. and international guidelines for research ethics with women, pregnant women, vulnerable subjects, and ethical guidelines for research in developing countries.

The following criteria were applied to a comprehensive list of ethical and social justice issues related to preterm birth and stillbirth to obtain a preliminary list of key gaps:

1. Extent and quality of discussion in the peer-reviewed literature

2. Degree of consensus on the ethical issues (represented in points to consider, clinical guidelines, domestic or international policy guidelines)

3. Scope of discussion, from low-, middle-, and high-income countries

4. Issue may impact basic science or development research on preterm birth and stillbirth

5. Issue may impact scale-up or delivery of interventions to prevent preterm birth or stillbirth

6. Issue may impact visibility and advocacy surrounding preterm birth and stillbirth

7. Issue may impact our understanding of the disease burden

The preliminary list was presented to the investigator team and SAC members for refinement. The following topics were identified as priority issues requiring future research and public deliberation according to the seven criteria above. To correspond with the scientific gap analysis, the topics are presented here as they arise along the translational pathway in preventing preterm birth and stillbirth, beginning with definitions and discovery, through development and delivery. Research questions identified throughout the analysis and in the deliberation of the Ethics and Social Justice working group are summarized in Table 1. These questions are intended to shape a bioethics research agenda in both social science and normative ethical analysis to inform a sustained and responsible program to reduce the burden of preterm birth and stillbirth within the broader context of improving women's health, as well as maternal, newborn and child health.

**Table 1:** Research questions: ethics and social justice

	Topic Areas	Research Questions for Social Science and Normative Ethics
Definitions and Measurement	Visibility:Global Burden Measurement Health Reporting & Data Collection	• To what degree are critical scientific definitions and classification surrounding preterm birth and stillbirth shaped by social and moral norms, and how do controversies over definitions affect visibility of the disease burden? • What are the psychological, social and economic costs associated with increased rates of prematurity in both HICs and LMICs? • How can we expand and improve global measures of stillbirth while avoiding implications for the abortion controversy?
Discovery Science	Research Ethics Community Engagement Improving Translation	• Are there additional ethical issues to consider in the design of biorepositories for the study of preterm birth, and how should these issues be addressed? For example, what are the attitudes and expectations of women who donate to biorepositories for the study of preterm birth and stillbirth? • What are the risks for stigmatization surrounding research on infection and preterm birth in vulnerable populations or marginalized communities? What is the potential ethical and social impact of the microbiome model of infection in the context of preterm birth? • What are the barriers to effective translation between discovery science research on preterm birth and stillbirth and the needs of women and families in LMICs? • To what extent can the prevalence of preterm birth or stillbirth be attributed to issues of racial, gender, or economic disparities and how can we target these systematic or structural causes?
Interventions	Expanding Outcomes Measures Socioeconomic Determinants Research Ethics	• What is the impact of maternal socioeconomic status on long-term outcomes for preterm births? • Can we estimate the family and social burden of improving preterm survival, a certain proportion of whom may go on to have significant problems? • How should these data inform intervention strategies? • What is the subjective experience of disability among preterm birth survivors (accounting for variation across socioeconomic status, culture, lifespan, and parental vs. provider perceptions)? • In the design of ethical neonatal intervention trials in developing countries, how can we avoid moral "double standards" in our choice of baseline interventions or control groups, while recognizing real limits to the resources available in low income settings?
Delivery of Interventions	Medical Decision-Making Women's Health Cross-Cultural Experience Health Equity	• In HICs, how should we balance women's reproductive choices and parental discretion against the impact and costs of preterm birth associated with the use of reproductive technology and fertility treatment? • What ethical dilemmas and value trade-offs do mothers, parents, families, and providers face during pregnancy and with preterm survivors, in settings where women and families lack economic or social safety nets? • What are the cross-cultural attitudes and perceptions regarding preterm births, stillbirths, and associated interventions, and how might these beliefs impact the acceptability of new approaches and treatments within a culture? • Can we identify better strategies and ethical guidance for balancing the implementation of short-term interventions while working toward more ideal, longer-term solutions in both maternal and newborn interventions? • How can we improve and further specify instruments for measuring impact on equity in preterm birth and stillbirth interventions?

## Results

### Ethical issues in definitions and measurement

Definitions of diseases, their causes, and chosen outcomes for research and interventions are shaped by the scientific community as well as social, political and ethical norms across populations and cultures. For example, "miscarriage," "stillborn," or "early fetal death" define cut-off points for viability. Medical determination of viability is based in empirical science but beliefs about the significance of viability are influenced by morality, culture and politics. Since measuring the burden of disease across and within populations is an essential tool for raising global visibility among those shouldering a greater disease burden, accurately measuring the magnitude and extent of particular diseases or health outcomes is a critical tool of practical social justice. If aspects of the global disease burden are poorly described, disease impact may be underestimated, and the suffering and social costs may remain unnoticed and unaddressed.

#### Measuring the global burden of preterm birth and stillbirth

##### Preterm birth

Measuring the global impact of preterm birth is confounded by different abilities to care for the preterm neonate and different expectations of when a neonate is considered viable. While the definition of preterm birth is uniform across HICs and LMICs (a birth at <37 completed weeks of gestation), the expectations of care for preterm neonates differ widely, and often as a function of inadequate health system and family resources. For example, in HICs a 27-week preterm neonate has an 80-90% chance of survival, with an approximate initial direct medical cost of US$150,000. In LMICs with limited neonatal intensive care resources and no public or private health insurance for families, a 27-week neonate may not be considered a candidate for resuscitation. Many who then die at this gestational age would not be counted as a registered death or stillbirth.

Encouraging uniform definitions of preterm birth is necessary for sound epidemiology and accurate assessments of the global burden. Consistency is also ethically significant. Continued variability in what practically counts as a "preterm worth saving" reveals and reinforces troubling health disparities. What should be a clinical and family decision is instead largely driven by a family's poverty. With this in mind, the ethical lesson from high-income countries is double-edged. What NICUs have made possible in high-income countries serves both as a high waterline for what is possible—that having a healthy preterm baby might one day cease to be a function of being born in a wealthy setting—and a cautious reminder that saving lives in the extremely preterm range carries significant costs for families and society and requires limits.

Inconsistencies in using low birth weight measurements versus gestational age are also in part a function of inadequate resources in LICs and rural areas, where the majority of births occur in the home, or in facilities with inadequate equipment or trained staff to gather and record such information at birth. In these cases, variation across measurements of preterm births not only poses a challenge for accurately assessing the global disease burden of preterm births, but signals underlying health disparities in preterm survival in resource-poor settings. Distributive justice tends to prioritize critical health outcomes, such as decreasing neonatal and maternal deaths. The significance of improving infrastructure for improved health measurement and vital statistics is often overlooked when considering moral arguments for redistributing scarce health resources. Funding agencies also typically target highly visible outcomes, such as reductions in mortality, to demonstrate direct impact due to funded interventions. While the results-based financing approach is central to transparent and efficient investment practices, it can nonetheless reinforce the lack of attention to longer-term investments in health systems. In the current debate surrounding the development of "diagonal," rather than vertical or horizontal investment programs in global health [2,3], it will be important to consider the relatively low-cost, high-impact investments in equipment and training tools needed to improve vital registration and gestational age measurements. Such investments would vastly improve the visibility of the preterm birth burden as a contributor to neonatal mortality.

Including causes in preterm birth population health measurements can be methodologically challenging but ethically significant. Careful calculations of distributive justice require careful population health measurement, including data on causes. An increase in preterm births as a raw measurement is not necessarily an indicator of poor population health or health disparities. For example, the percentage of preterm births in HICs that are medically indicated preterm deliveries, due to a distressed mother or fetus, may actually signal a well-funded health system and quality perinatal care for high-risk pregnancies. As middle-income countries build NICUs and improve access to facility births, moderate rises in medically indicated preterm births should not necessarily be a cause for alarm. While preterm births caused by untreated infections or maternal malnutrition would likely represent a failure of access in an HIC, they would represent a funding or capacity-building gap in an LMIC. From the vantage of public health ethics, there is also a need to better understand the causal links between preterm births and occupational, environmental and personal health risks, such stresses in the workplace or smoking. Research on global environmental risk factors is needed to help guide public health interventions motivated by claims of social or personal responsibility.

In the clinical context, consistent use of cutoff points for viability in preterm births is complicated by varying beliefs regarding moral significance of those cutoff points. There is a fairly extensive discussion in middle- and high-income countries surrounding the ethical significance of thresholds of viability and the definitions of "prematurity" and "extreme prematurity" [4,5]. This discussion remains controversial. While it is useful to defend a clinical cutoff point for viability, this will not obviate the need for addressing those parents or communities viewing this as a moral or religious question, regardl ess of medical opinion or data on clinical outcomes. In this case, lack of a clear consensus on definitions impacts a range of clinical decisions: fetal surgery, resuscitation, limitation or withdrawal of treatment, palliative care and pain management, and cutoff points for other life-saving interventions.

##### Stillbirth

A number of perplexing moral questions arise in the measurement of the disease burden associated with stillbirth. In global mortality statistics, stillbirths are still largely unreported as deaths [6,7]. WHO estimates that of the 10.6 million child deaths before age five in 2001, 3.9 million occurred before 28 days. Another 3.3 million stillbirths were not included in the vital registration systems [8,9]. Health system limitations as well as economic and political barriers contribute to underreporting. There remain cultural and moral questions that may impede efforts to improve estimates of stillbirths worldwide. Making these issues explicit may help health officials adequately address cultural and ethical debates. Debates over ambiguous information may risk derailing positive efforts to address this high impact issue.

"Stillbirth" is not a well-defined term, but a colloquial term that is often used inconsistently. Confusion over the definition of stillbirth, intrapartum death, and miscarriage, when collecting reports in and out of facilities, makes consistent registration challenging. As discussed in the article 1 of this report, stillbirth may refer to late fetal death, which is a death after 28 weeks gestation or at least 1000 grams birth weight, or it may include early fetal death, which is a death after 22 weeks gestation or at least 500 grams birth weight. Thirty years ago, the minimum gestational age for classification as a stillbirth was 28 weeks. The definition has become progressively inclusive by decreasing the minimum required gestational age. These different measures of stillbirth have had a significant impact on infant mortality estimates. The official definition of a stillbirth in the former Soviet Union, for example, led to a 20-25% underestimation of the infant mortality rate:

"Babies who were less than 28 weeks, even if they showed some signs of life (breathing, heartbeat, voluntary muscle movement), were classified as 'live fetuses' rather than 'live births'. Only if such newborns survived seven days (168 hours) were they then classified as live births. If, however, they died within that interval, they were classified as stillbirths. If they survived that interval but died within the first 365 days they were classified as infant deaths [10,11]."

In the most basic sense, to count a death is to grant significance to the life lost. How to count a death and whether to count a death is then informed by our beliefs about the value of what has been lost. This value drives the justification for preventing similar future losses. Several moral and cultural challenges remain beyond the methodological challenges of measuring stillbirths and the associated health burden. Politically, in some countries, counting early fetal deaths as deaths implies that a life worth preserving was lost, and therefore, that life at 22 weeks gestation, and perhaps earlier, is a life that should not be deliberately taken. This has obvious implications for the abortion debate, and some countries have resisted including stillbirths among vital registration or mortality statistics for this reason. For the purposes of improving vital registration and decreasing stillbirth rates, it is worth encouraging a frank discussion about this question to either find common ground or to find ways around the moral impasse. Of particular concern worldwide is avoiding direct or indirect impact on the reproductive rights of women by unintentionally fueling the abortion debate. This could be a case where well-meaning health policy aimed at improving the health of women and neonatal survival has an additional uni ntended consequence of impacting women's reproductive choices, particularly in those countries where reproductive freedom remains tenuous.

This debate potentially affects how we measure the loss of a stillborn within global vital registration systems used to allocate health resources. For child and adult deaths we can record the age at death, the cause, years of life lost (YLL), and the years of healthy life lost due to disability— also known as Disability-Adjusted Life Year (DALY). In addition to lacking sufficient data on causes for stillbirths, measurements of deaths near the time of birth present a challenge due in part to puzzles over how to consider the life lost [9]. When including stillbirths in global burden of disease estimates, it remains unclear whether the best approach is to incorporate stillbirths within DALY measures, using infant death equivalence or life years lost. That is, should the loss associated with a stillborn be measured in terms of the lost "potential life lived," and if so, how could such a counterfactual measure be made meaningful? And if infant or adult-death equivalences are used, how should the loss of a stillborn baby be discounted? That is, by what percentage is a death of a stillborn less important than a death of a newborn at term, versus a child, versus an adult? Addressing such questions is not merely a philosophical exercise, but is necessary to develop disease burden measurements that accurately represent the magnitude of the burden.

The current measurement model used by the GBD and many country estimates lists stillbirths as a parallel statistic, without trying to incorporate stillbirths into DALYS. Using parallel measures risks the burden not being taken as seriously in resource allocation decisions, typically linked to GBD measures, requiring special interest groups to lobby for attention to stillbirths. If integrated into DALYs or other aggregate measures, stillbirths are more likely to be addressed equally with other contributing factors to disease burden in neonatal, maternal, and population health. Including stillbirths as a function of the mother's DALY would avoid the puzzles in measuring the loss of a stillborn. However, this method yields very low numbers and is not considered an accurate measure of the global burden of stillbirth. It is worth considering whether we can develop better measurements of the psychological and health impact of stillbirths on women (for example, depression or impact on fertility).

In summary, several points can be made to avert entanglement with the abortion debate and to focus instead on the straightforward value of increasing the visibility of the disease burden associated with stillbirths, with improved vital measurements of stillbirths. At first glance, it seems that those who endorse arguments for the intrinsic value of preventing early fetal deaths must accept certain implications regarding the moral permissibility or impermissibility of abortion at viability. However, there are a number of compelling reasons for preventing stillbirths, none of which invokes abortion:

1. Stillbirth is a significant contributor to the disease burden of women and a marker for poor population health. Decreasing stillbirths is an important means for improving maternal health; understanding the causes of stillbirths may help address broader determinants of poor population health.

2. The causes and circumstances of many stillbirths are reasonably preventable—stillbirths of pregnancies due to maternal malnutrition or syphilis, for example. Stillbirths that would have resulted in healthy term births, but for a preventable maternal infection or inadequate prenatal care, are worthy of prevention according to most criteria of social justice. Intrapartum stillbirths are especially associated with maternal morbidity and mortality. These represent 30% of all stillbirths and are typically preventable.

3. Efforts to decrease stillbirths are aimed at preventing what are presumably, in most cases, deaths of wanted pregnancies.

4. We should distinguish intrapartum stillbirths from early intrauterine deaths in the global burden of disease measures, thereby making important incremental gains by including intrapartum stillbirths in global estimates, on par with neonatal deaths. Even those who deeply disagree about when life begins will concede that it is morally arbitrary to hold that a gestationally viable infant who dies during delivery does not count at all, whereas an infant that dies after being delivered and taking one breath, counts as a neonatal death. This is not to underestimate the challenges of determining the loss associated with gestationally early antepartum stillbirths. The latter problem requires targeted and interdisciplinary deliberation, particularly on the issue of unintended impact on women's reproductive freedoms.

For investigators and funding agencies straightforwardly interested in decreasing the obvious disease burden of stillbirth while sidestepping these difficult moral questions, the most direct appeal may be made to the value of promoting women's health through the decrease of stillbirths, and by promoting the value of healthy births at or near term.

#### Measuring the social, psychological, and economic impact of preterm birth and stillbirth

Missing from the global measures of both preterm birth and stillbirth is a thorough account of the subjective experience of the loss to mothers, parents, and communities. While we have some data on the psychological experience of stillbirth and preterm birth on mothers and fathers in HICs [12-15] we have little understanding of the cultural and ethnic variation among such experiences, or the magnitude of the psychological burden on minority or marginalized populations in HICs or LMICs.

Social values inform the different labels assigned to a pregnancy that do not result in a live birth. With a stillborn, cultures may support rituals of grief and mourning for the mother or parents, such as naming or a burial, yet such rituals are not typically available for a miscarriage when the loss may be equally strong in both parents [16]. In Pelotas, Brazil in the 1980's, stillborn babies were put in the "next available" coffin with a dead adult and buried together. This would save the families the cost of a funeral, and the hospital staff did this routinely. As a result, those deaths went unreported for many years, until the hospital staff and undertakers were persuaded by the local epidemiologists to stop this practice and to begin recording these deaths (Victora and Barros, personal communication). A "miscarriage" can also imply inadequacy in the mother, as do repeated preterm births or stillbirths [16]. In some cultures women who have stillborns or early postnatal deaths due to preterm birth may be socially stigmatized by their husbands, required to undergo cleansing rituals, accused of infidelity, or divorced [17]. Additional social science research is needed to better understand the social and psychological aspects of the disease burden, and to improve support interventions to be implemented alongside interventions aimed at prevention.

Cultural beliefs and socioeconomic status may also impact reporting preterm births and stillbirths. In populations where high infant mortality rates and high stillbirth rates are common, a degree of fatalism may impact the experience and reporting of fetal or neonatal loss. These are general challenges in measuring the more elusive social and psychological impact of disease in country-level and global measurements, but there remains a specific research gap for social science researchers interested in capturing the cultural and social barriers to reporting both preterm birth and stillbirth.

In HICs where rates of preterm birth may be due in part to increased use of Assisted Reproductive Technologies (ART) and fertility treatments, measuring the long-term social and economic costs of unregulated use of these technologies is a critical step toward decreasing preterm birth-related morbidity. In countries like the United States where such choices remain largely unregulated, little is known about the wider impact of preterm births on the health and education systems, and on families. In a culture that favors the freedom of choice, such health and economic measures would provide an important correction to the moral balance between maternal and parental choice, preventing harm to others, and the social responsibility of parents and providers.

Stillbirth and preterm birth are important indicators of child health, women's health, and social and economic inequalities. For this reason, addressing the measurement gap is itself an important instrument of social justice. Improving methods for measuring rates of preterm birth and stillbirth globally and addressing the cultural and ethical beliefs surrounding deaths near the time of birth will help address a significant blind spot in appreciating the complex and significant global burden of these outcomes.

### Ethical issues in discovery science

Few ethical issues in discovery science related to the causes and prevention of preterm birth and stillbirth are unique to these outcomes. Most have been thoroughly discussed in the literature on basic science research ethics. There are three emerging research areas to prevent preterm birth that raise special concerns. The first is the ethical design and use of biorepositories for the purposes of studying preterm birth, the second involves microbiome, genome, or genetic analysis in the study of infections and preterm birth, and the third involves the more general challenges of improving clinical translation from basic science research in HICs to use in LMICs.

#### Biorepositories to study preterm birth: informed consent and returning results

There are two familiar questions in discussions surrounding governance and guidelines on biobanking: How can an ethical plan be designed to return results to biobank participants and/or a study community? And, how can robust informed consent be ensured for participants [18-20]? A number of repositories are being established to study the causes of preterm birth. Such repositories will enroll pregnant women, as well as the first and second degree relatives of women who have experienced preterm delivery. Biological samples and clinical information are collected and typically de-identified to protect the confidentiality of individual participants. Most studies will be conducted by secondary investigators, who are granted access to repository samples. As with any other biobank, the scope of institutional and investigator responsibility to disclose specific, aggregate, or incidental findings to individual participants (where linked), or participating communities, will need to be established in collaboration with IRBs and community stakeholders.

The rise of genome wide association studies has reinvigorated a complex debate that began nearly two decades ago regarding the duty to disclose individual genetic research results to participants, beyond sharing aggregate results in the form of publications or research summaries [21-24]. The main considerations supporting non-disclosure of individual results have been two-fold: If a research study is designed to produce general knowledge and is not expected to benefit individual participants or expected to yield clinically significant findings, then the returning results would run contrary to the aims of non-therapeutic, basic science research. Further, returning results may unnecessarily harm participants if they make significant health decisions based on highly uncertain, invalid, or poorly interpreted data. However, disease advocacy groups, bioethicists, and expert policy and regulatory groups have joined in an international push for more nuanced guidance based on a full range of considerations, including the rights and interests that participants may have in knowing individual findings, concerns about genetic confidentiality, as well as the potential impact of population-based genetic studies [25,26]. There is currently fair consensus on the importance of disclosing only clinically validated results and developing a contingency plan for disclosure in partnership with an IRB. However, there continues to be serious disagreement about the specific criteria for disclosure and clinical validity [27].

As this debate continues and guidelines for disclosure are further developed, researchers and IRBs will need to make decisions regarding the plans for disclosure and the sharing of research results. During informed consent at the time of collection, it will be crucial to clarify whether the team plans to return results, whether participants may request or decline individual findings, and whether samples may be withdrawn at any time. If downstream clinical applications are not expected for several years, study teams should consider offering interventions or information that will facilitate healthy pregnancies or postnatal care for participants at the time of enrollment. As results become available, the usual precautions should be taken when returning individual results to clarify clinical significance and to offer genetic or clinical support and counseling to help explain the findings, and to assist with the decision to inform or not inform relatives. Sharing aggregate results may be facilitated by password-protected web-based research updates and newsletters, to keep participants aware of ongoing aggregate findings. This can include contact information for participants with questions about the study and its findings. Such a site can also serve as a place for posting educational information about healthy pregnancies and additional resources for participants. Because of the psychological burden that attends preterm birth for women and parents globally, biobank institutions may be in a position to facilitate social networking among participants by taking extra steps to design a biobank website that allows for participant-to-participant contact, or anonymous posting to a blog. This is a way to engage participants in long-term studies and to provide benefit, when significant clinical findings (and therefore direct benefit) for individual participants are not expected.

Participants and study communities must consider the validity and clinical significance of the results as well as its personal meaning of the results to participants and study communities. The obligations to return results will depend upon the scope and duration of the relationship between the repository institution, investigators, and participants. In the case of studies targeting particular communities, especially ethnically-defined communities, the scope of obligations may be extended to the relevant study communities. Care should be taken during community engagement to discuss how the research findings will improve the health of the community in the long-term, and how concerns about potential stigmatization will be addressed.

These are familiar ethical questions in biobanking research in general. What preterm birth research may add to the ethical considerations is an additional concern for the expectations and experience of participants. Women or couples who have suffered through one or more preterm births, pregnant women who have experienced a prior preterm or stillbirth, or have a sister or mother who experienced preterm delivery, will likely experience heightened anxiety about their pregnancy that should be taken into consideration during the recruitment process. Similarly, such women may have an expectation that by participating in such research, they will "find a cure" to prevent preterm delivery for this pregnancy or the next one [28]. Attention to these issues should shape community engagement, the informed consent process, and the eventual return of results.

#### Microbiome and genome research surrounding preterm birth

Like the Human Genome Project, the goal of the Human Microbiome Project in the United States is to characterize the human microbiome and create a technological and data-rich resource that will enable in-depth study of its variation and its influence on health and disease. For both genomic and microbiome research an important target area for application of these data is in the field of preterm birth, to understand the role of the human microbiome in the perinatal period. A recent study concluded that the amniotic cavity of women in preterm labor harbors DNA from a greater diversity of microbes than previously shown, including previously uncharacterized taxa, suggesting a causal relationship [29]. These data will likely lead to efforts to characterize the microbial signatures of healthy and preterm amniotic cavities, in an effort to reduce preterm labor and preterm birth. As this research moves forward, it will be important to remember that this new genetic frontier lies within individual women, who may have a range of questions, concerns, and needs that should be carefully considered in study design and follow-up.

Research applying human genomic or microbiome project findings to preterm birth raises several familiar ethical and social issues in new ways. Future results may challenge historical definitions of "contamination," "contagion" and "infection," by detecting the presence of previously unknown microbial species, and by detecting microbes not detectable with non-genomic techniques. There is potential for individual or group stigmatization surrounding demonstrated or suggested causal links between infections, especially sexually transmitted infections, and increased risks for preterm labor or stillbirth. History on the impact of infectious diseases in already marginalized populations, such as syphilis or HIV, gives reason for caution in disseminating such findings [30].

The burden of preterm birth is often greater in racial/ ethnic minority groups, such as black women in the United States or women of lower socioeconomic status globally, with poor access to prenatal care and facility births. For this reason results of microbiome research in particular, and research on the links between infection and preterm birth in general, may disproportionately affect certain communities of women who in turn may have little opportunity to gain from the technological benefits of the research. Further, both genome and microbiome arrays rely on techniques that allow for the detection of a very broad range of genetic information, only some of which is significant to understanding preterm birth, but with very uncertain downstream applications for current research participants. Retrospective genomic and microbiome research, beginning for example with developmental delays in preterm survivors and returning to sequence the mother's and baby's samples, will require similar consideration of the impact of these findings in the informed consent and dissemination processes.

#### Improving translation between discovery science in HICs and delivery in LMICs

Despite the global impact of preterm birth and stillbirth, in terms of lives lost, psychological impact, and long-term economic impact of the morbidities associated with preterm survivors, the research agenda for the prevention of preterm birth remains significantly underfunded compared to other contributors in the global disease burden. As global visibility of the disease burden associated with preterm births and stillbirths improves, one of the emerging challenges will be to improve translational applications of discovery science research to LMICs [31] and to improve investment in discovery research capacity and investigators in LMICs [32].

**Figure 1:**
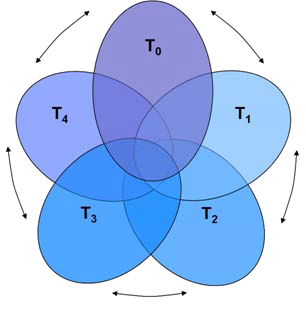
**Expanded translational model.** Source: Starks *et al*. [33].

Efforts are underway to improve the process of clinical translational research—research moving basic science research to effective clinical interventions—ensuring that public investments in basic science research lead to improved individual and population health outcomes (Figure 1). New, in this analysis, is the concept of a pre-pathway period, designated as T-0, on the standard T-1 to T-4 stages of biomedical research, where:

• T-0 phase represents the period during which research problems and opportunities are identified,

• T-1 represents discovery science, where opportunities to improve health are identified and pursued,

• T-2 the development of potential health applications to practice guidelines,

• T-3 the delivery of health applications in practice,

• and T-4 the evaluation of outcomes and impact [33].

A key component of the expanded model reveals the often implicit value judgments that inform the choices among and within research agendas. Some value judgments reflect important, though often implicit, deliberation about the just distribution of scarce research resources. The expanded model of translation attempts to make such value judgments more transparent [34].

With the expanded translational model in mind, a T-0 challenge in preterm birth research, prior to T-1 setting basic science research agendas and funding, is to ensure that LIC needs in neonatal and maternal health are informing discovery science research agendas in HICs. In HICs, incentives shaping research agendas are still largely driven by academic advancement within HIC universities, economic incentives in private industry, and politically powerful stakeholders in health funding and investment. Ensuring feedback and input from study communities, especially in LMICs, requires a cyclical view of the translational process, as opposed to the standard linear, or "pipeline" view [33]. Improving discovery science translation for LMICs will also require creative thinking for implementing promising systems biology research in these settings. Those engaged in systems biology research in preterm birth might look to the examples of HIV, TB, and Malaria research conducted in LMICs where strategies have been developed to address barriers to training, consistent collection techniques, and adequate storage capabilities.

Finally, improving translation on preterm birth research from low-income to high-income settings is a pressing issue for research priority-setting within HICs. Women living in poverty and women from underserved ethnic or racial groups in the United States, for example, suffer higher rates of preterm birth and infant mortality [1,35]. Such data reinforce the need for a research agenda that is shaped by the needs of underserved populations wherever they may be found.

### Ethical issues in development of interventions

Developing interventions to decrease the burden of preterm birth and stillbirth will involve identifying effective interventions along the continuum—from maternal health before and during pregnancy, labor and delivery, to care of the newborn and mother after birth. Ideally, development of interventions will investigate the underlying disparities and conditions that contribute to high rates of preterm birth and stillbirth in certain populations. The combined goal is to improve the health of women, improve preterm survival and subsequent morbidities of preterm survivors, and to reduce still-births—with special attention to addressing underlying health disparities across this continuum. However, taking such a holistic approach to maternal and child health in the context of preterm birth and stillbirth risks defining the problem so broadly that it may be difficult to make progress on any particular outcome, such as increasing preterm survival. Study design and implementation for the development of effective interventions in LMICs must also address the constraints of limited resources, participant and population vulnerability, and disease prevalence unseen in HICs. Most challenging will be to determine what can be done now to address the disease burden in a particular context while not losing sight of long-term goals of equity in disease burden across and within HICs and LMICs. A number of ethical considerations arise in development science in preterm birth and stillbirth over these issues of investigative scope and the need to balance scientific rigor with immediate versus long-term impact.

#### Morbidity and outcomes, beyond survival

Improving the lives of children by reducing childhood mortality, illness, and disability requires a better understanding of the causes and long-term outcomes associated with preterm birth. In high-income countries, especially the United States, focus on improving the survival of preterm infants continues to eclipse the prevention of preterm birth. Heralding records for lower gestational weight infant survival or successful deliveries of multiple births following assisted reproductive interventions, risks diverting attention from a critical blind-spot regarding the impact, cost, and challenges associated with increasing rates of preterm births. Those who survive preterm birth and low birth weight have increased health risks, including blindness, cerebral palsy, behavioral and attention deficits, and chronic lung disease. A number of these health risks have lifelong impacts on the children born preterm, and families and communities who care for them. The burden of such outcomes is significant for families who live in poverty and lack resources to support children with special needs. This gives an even greater impetus for identifying and implementing effective interventions for preventing preterm births and stillbirths in low-income communities. It also reveals the importance of considering the long-term consequences of intervention programs. Responsible intervention programs aimed at improving neonatal survival will address the broad range of community concerns for improving neonatal and pediatric care and services, if not directly, then through parallel partnerships that address structural and health systems needs.

Preserving and promoting quality of life requires continued research on the long-term outcomes of preterm survivors. A number of ethical issues need to be addressed when choosing outcome measurements in the design of intervention studies: (a) which criteria should be used to measure long-term morbidities and quality of life among preterm survivors, (b) should we consider the availability of diagnostic/intervention services for survivors of preterm birth with impairments when implementing life-saving interventions during the neonatal period, (c) what is the impact of maternal socioeconomic status on long-term outcomes for preterm birth, and (d) what is the subjective experience of disability among preterm birth survivors (accounting for variation across socioeconomic status, culture, lifespan, and parental vs. provider perceptions).

In HICs recent ethical discussions focus on the responsible use of assisted reproductive technology (ART), a contributing factor in the rate of preterm birth in the United States. Despite the link, little attention is given to long-term preterm survivor support as a consequence of ART, or the value trade-offs that parents must make, such as weighing the benefits of delayed reproduction with increased risk for preterm birth. Also lacking are reliable estimates for the socioeconomic impact of preterm and multiple births associated with fertility treatments and ART—the impact on health systems, educational systems, families, and survivors. Given the value of child survival and the importance of preserving and promoting quality of life, addressing the long-term outcomes of preterm survivors is critical across borders and economic lines.

Similarly, in HICs there is a tendency to focus intervention efforts on increasingly sophisticated treatment at the time of labor or treatment of the preterm infant, and relatively little attention to clinical or public health interventions on means of prevention for preterm birth or stillbirth. This may represent the broader tendency in many HICs to invest in treatment over prevention—a possible bi-product of a health system driven by consumer demand. Cultural norms favoring individual choice over personal and social responsibility may also contribute to the focus on intervening at the point of delivery and preterm birth, since public health or behavioral interventions may be viewed as impeding free choice.

#### Designing ethical intervention trials in developing countries: The "double-standards" debate

In most parts of the developing world, babies are born at home and neonatal care is not available to the majority of these newborns. Neonatal mortality rates are high in these countries, with preterm birth, birth asphyxia or injury, and infections representing the leading causes of death. Controlled trials on potential neonatal interventions are essential to address the direct disease burden in poor communities and the underlying disparities that have led to such disproportionately high rates of infant mortality in LICs. When attempting to develop interventions that will be sustainable within local economic constraints and socio-cultural conditions, researchers, sponsors, local and international IRBs, and communities continue to face one of the most vexing issues in international research ethics: When the standard of care varies within and between countries given resource constraints, which standard of care should serve as the baseline for determining which interventions can be tested in a study and what, if any, interventions are offered to participants in a control group?

To illustrate the issue, consider a landmark trial conducted in Gadchiroli, India, to address one of the leading causes of neonatal mortality in rural India: neonatal sepsis [36]. While the standard of care for neonatal infections in high-income settings is neonatal intensive care and expensive intravenous antibiotics, such interventions are not readily available in urban centers in India much less in the very rural districts. Was it possible to develop lower-cost, safe, and effective interventions for infants in areas such as Gadchiroli? An earlier study of pneumonia management in neonates using cotrimoxazol administered by community-based health workers had demonstrated a 20% reduction in neonatal mortality. Based on these data, researchers in the Gadchiroli trial developed a package of home-based neonatal care that included the management of neonatal sepsis, meningitis, and pneumonia. Investigators hypothesized that this package of interventions would be safe, feasible to administer in the field, and could reduce neonatal mortality rate by at least 25% in 3 years. The cluster-randomized intervention trial was conducted over 5 years in 86 villages in rural India, including 39 intervention villages, and 47 control villages. The intervention areas and control areas each included about 40,000 people. In the 47 control villages, a baseline survey was conducted, and standard interventions offered. In the 39 intervention villages, female village health workers were trained in the home-based management of neonatal illnesses and provided with a care kit that included basic medical supplies, supplies for infection control. For newborns suspected of having sepsis, health workers administered oral trimethoprim-sulfamethoxazole and gentamicin. Health workers in the intervention villages tracked pregnant women, observed labor and delivery, and visited the home on days 1, 2, 3, 5, 7, 14, 21, 28, and any other day the family called in order to examine the baby, weigh the child, and treat minor illnesses, sepsis, pneumonia, and meningitis [36,37]. Community consent was obtained from the intervention villages and the study was subject to an extensive scientific and ethical review process involving national experts and the Indian Council for Medical Research. With this approach, Bang and team demonstrated a 72% reduction in neonatal mortality, a result that continues to impact national and international approaches to neonatal care in low-income settings [38].

And yet, the study came under scrutiny for the presence of control villages where only the local standard of care was offered, since not even the national standard of care was considered sustainable [37]. Consider the principle put forward in the Declaration of Helsinki, which states, "In any medical study, every patient— including those of a control group, if any—should be assured of the best proven diagnostic and therapeutic method [39]." On a strict interpretation of this principle, it would be impermissible to allow the study of an intervention significantly beneath the best standard of care, presumably in the Gadchiroli trial, neonatal intensive care and frontline intravenous antibiotics [40]. Under a strong interpretation of universal standards, this trial would never have been approved and thousands of infants would have died who were otherwise saved. For contexts where there is an urgent need for developing low-cost, effective and locally sustainable interventions, others have argued for a more reasonable interpretation of the Helsinki principle that allows for consideration of regional context and participant needs when determining the ethically appropriate standard of care, while maintaining universal standards against clearly exploitative research with vulnerable populations [38,41-43].

The more profound question regarding the standard of care debate is how to pragmatically address urgent local needs while not losing sight of higher aspirations to address the vast health and economic disparities that will continue to fuel the gap between rich and poor in neonatal and maternal care. That is, how can immediate research needs be met in accordance with local standards, often saving lives and reducing disease burden that would otherwise go unaddressed, while ensuring long-term solutions are pursued? The investigators in Gadchiroli and other community intervention studies offer laudable examples of how to establish long-term relationships with community leaders, study participants, and the broader communities to ensure ethical study design that is sensitive to the local context, while continuing to fight for improvements in health for all on the national and international stage.

#### Health disparities and the determinants of preterm birth and stillbirth

A sustainable approach to preventing preterm birth also requires attention to the systematic causes of preterm birth, such as socioeconomic determinants of maternal health and healthy pregnancy. Important work has been done on the social determinants of health in general. There is robust emerging literature on the social determinants of race for preterm birth [44]. Additional research is needed on potential solutions to continue mapping the global social and economic determinants of preterm birth and stillbirth. There are emerging but conflicting data on specific causal links between types of maternal stress and preterm birth, such as depression or stressful life events [45,46]. This remains a significant research gap in LMICs and for women living in poverty or women of racial-ethnic minorities in HICs. One study found Black women and American Indian/Alaska Native women to have a significantly higher rate of stressful life events in the 12 months before delivery, although this racial-ethnic disparity in the experience of stress did not contribute significantly to the racial-ethnic disparity in preterm birth [46].

Targeting the underlying health disparities in maternal health that lead to higher rates of preterm birth and stillbirth is important for the sake of women's health. More than a half million women continue to die each year in childbirth, often due to inadequate resource allocation in maternal health and a complex web of social determinants, inadequate clinical capacity, and health disparities [47]. Stillbirths are an important indicator of maternal health disparities. Measuring the counter- factual—"these would have been live births if proper maternal care had been available"—is challenging but crucial from the point of view of distributive justice.

Promoting women's health, literacy, education, and reproductive choices (including contraception and birth spacing) are intrinsically valuable, and are also important means for lowering rates of preterm birth and stillbirth. Given the intimate connection between women's health and the prevalence of preterm birth and stillbirth, sustainable interventions will target women's health and socioeconomic well-being, not merely women's reproductive health or child survival. Developing effective interventions to ease the global burden of preterm birth and stillbirth should be the aim of scientific investigators, but social science research, public policy, strategic long-term funding and advocacy have an opportunity to address the underlying social and political causes fueling this and other aspects of the disease burden in women's health, neonatal survival, and child health beyond survival.

### Ethical issues in delivery of interventions

Effective interventions to prevent preterm birth or stillbirth already exist, or have been shown to be effective in improving preterm survival, but fail to be implemented where needed most. Many such interventions, such as antenatal steroids or antibiotics, are available at a relatively low cost. In this way delivery barriers are in some ways more tragic than having no effective interventions at all. Experts may debate and refine principles of justice to prioritize effective interventions but if social, political, or economic barriers prevent implementation, this represents a failure of practical distributive justice. Concerns of social justice are thus intimately tied to a rigorous approach to delivery science and a delivery gap analysis [48,49]. Important efforts have been made to develop normative frameworks for health priority-setting in general [50]. While there is room for debate over principles, immediate and significant challenges remain in the practical scale-up of interventions and in the measurement of impact on equity for specific diseases [51,52]. These challenges arise more generally in all global health efforts, but it is worth highlighting the particular challenges that arise in the prevention of preterm and stillbirth.

#### Women's social and health disparities as barriers to delivery of interventions

High rates of preterm birth and stillbirth are not only markers for poor population health, but should be considered sentinel markers for social and health disparities facing women in these populations. Among the estimated 3.2 million stillborn worldwide, a significant number may be due to common, preventable causes, such as treatable maternal infections like syphilis, asymptomatic bacteriuria, or intrauterine malnutrition [53,54]. In particular, intrapartum stillbirths—30% of all stillbirths—are strongly associated with maternal morbidity and mortality. Access to prenatal screening for high-risk pregnancies, access to emergency obstetric care for obstructed labor, and treatments for infection or malnutrition often influences pregnancy outcomes (article 4 in this report [55]). More importantly, barriers to safe pregnancy interventions, simple screening, and antibiotics, are severely impacting women's health and lives [56]. Women's socioeconomic status is a known predictor of preterm birth and stillbirth, among other health outcomes. Literacy, education, and decision-making empowerment are known to improve women's access to prenatal, delivery, and postnatal care and affect women's reproductive choices, such as contraception and birth spacing. These choices reflect a demand for care that is necessary once interventions are made available. Lack of demand may be a sign of deeper social and political challenges facing women in these communities. For these reasons, partnering with local organizations to address women's social and health disparities and investing in health systems are integral to promoting women's health and human rights, as well as a necessary step in overcoming barriers to delivery of effective preterm birth and stillbirth interventions [47,57,58].

Within societies where women do not enjoy equal social and political status with men and lack social and political voice, demand for health services before, during and after pregnancy can be seriously reduced. Further research is needed to understand women's specific concerns about making decisions, traveling to a doctor alone, being seen by a male doctor, or disclosing sensitive information about sexuality. Because sensitivities and concerns about stigma or a husband's reaction may impede the scale-up of infection screening programs, such as syphilis screening, there is a need for social science research into the context-specific and cross-cultural concerns facing women when choosing to seek or not to seek screening and treatment.

#### Balancing short- and long-term outcomes: compromises vs. ideals

Maternal and newborn advocacy groups can be instrumental in addressing resource barriers to improved delivery of maternal and newborn interventions. However, these advocates do not always see eye-to-eye on the best strategies for achieving short- and long-term outcomes, especially when maternal health outcomes and newborn interventions seem to be in conflict. Using the example of emergency obstetric care, consider two different approaches to the problem. From a maternal health perspective, ideally, all women would have access to quality health facilities for safe deliveries at any risk level, so in the event of an unforeseen complication an emergency C-section is immediately available that could mean the difference between saving the life of the mother and baby, and losing both. Maternal advocacy groups have therefore promoted facility delivery for all women [59] and argued that investing in improving the quality of home delivery care—either by traditional birth attendants (TBAs) or medically trained birth assistants—contributes to delayed universal facility care. In most developing countries, it will take more than a decade to build the infrastructure and skilled staff necessary for attaining such universal access. In the meantime, women continue to give birth in the home, where many newborn lives and some maternal lives may be saved by cost-effective interventions that include training of TBAs and/or medically-trained health workers. Some newborn advocacy groups, therefore, have taken a counter position, arguing for the scale-up of such home-based strategies in settings where access to facilities is poor while promoting improved quality for care at referral facilities. However, because the best approach for ensuring maternal health is to improve access to resource-intensive, skilled emergency obstetric care, some maternal advocacy groups resist this less-than-ideal solution.

This example illustrates not only the challenge of balancing newborn approaches with maternal approaches, it represents a broader challenge in global health, namely, the need to address immediate and short-term needs with non-ideal interventions against longer-term needs with more ideal solutions. Holding out for the ideal interventions and health systems improvements may mean that present needs go unmet by those unwilling to scale-up interventions perceived as being less than ideal. However, short-term, compromise interventions have a tendency to become the accepted, standard intervention, given the challenge of shifting behavior, training programs and health resources. And the fear, perhaps represented by maternal advocacy groups, is that short-term interventions may undercut the political and economic leverage needed to make much more difficult, sustainable changes in health systems for women.

Further, the controversy over task-shifting reveals the tensions between professional stakeholders in maternal and child health. In many countries, both physicians and nurses unions and professional groups have been strongly opposed to, for example, teaching C-sections to technicians or allowing community health workers prescribe antibiotics for newborn sepsis, arguing that the quality of care will be inadequate if not outright dangerous. This issue has in part been addressed with empirical data suggesting that task shifting for C-sections [55,58,60-62] and antibiotic prescription by CHWs [63,64] have more advantages than disadvantages, but it remains an important ethical challenge in the delivery of short- and long-term global health interventions for maternal and newborn health.

#### Poverty and cross-cultural experiences of preterm birth and stillbirth as barriers to delivery of interventions

At the systemic and structural level, many of the most significant barriers to the delivery of effective interventions for reducing preterm birth and stillbirth reside with the health systems and ministries of health. However, solutions to these barriers often reside with more powerful areas of government such as the ministries of finance and foreign affairs [55,65]. In many LMICs, political instability and inadequate rule of law contribute to corruption and lack of accountability in the efficient and timely allocation of health resources to support maternal and neonatal health [66]. This is a challenge for delivery in any context, but within governments or health systems that do not value women and children as equal citizens, efficient delivery may be thwarted by a lack of political will rather than a lack of sufficient funding. These issues reflect a research and policy gap in institutional and health systems design, but also a recalcitrant problem in distributive justice and health promotion. The health and human rights movement offers a helpful framework for recasting delivery barriers as human rights violations, which can be used as leverage among international stakeholders. Recently this framework has been applied to the challenges of maternal and neonatal health, although it has not been applied to preterm birth and stillbirth specifically [57]. However, additional work is needed to address issues such as the impact of international loan restructuring and negotiated restrictions on government social spending. Addressing these deeper issues will require a critical and multi-disciplinary discussion about the design of just social and political institutions and norms supportive of complex global health efforts.

Poverty and rural geography also impact delivery of effective and often inexpensive interventions. Families living in rural areas of both LMICs and HICs have poor access to health facilities. As discussed, one of the major causes of neonatal and maternal mortality in delivery is the lack of access to emergency obstetric care, making simple geography a critical barrier to delivery of effective interventions like emergency C-sections. Accessibility also impacts availability of the trained staff, drugs and equipment needed to support complicated pregnancies. Populations living far from urban centers or in extreme poverty are also impacted by poor access to health information systems for disseminating information on nutrition, and to interventions supporting healthy pregnancies and safe birth practices (see Table 1 in article 4 of this report [55]). Advanced technology, mobile health communications tools, and health systems software are no longer viewed as tools of HICs. Instead, novel solutions for spanning distance with newer and cheaper information technology are becoming critical tools in practical social justice, mitigating the disadvantages of rural geography and the isolation of poverty [67].

As mentioned previously, a significant gap remains in understanding the personal, social, and cross-cultural experiences of preterm birth and stillbirth among women throughout the world. We do not have a clear understanding of women's, parents', and communities' experience of preterm birth and stillbirth. These outcomes are needed to have a complete understanding of the burden of disease and to shape culturally appropriate and gender-appropriate interventions. While some social science work has been done on provider's experience surrounding decision-making and preterm birth, and women's experience of miscarriage, very little work has been done to explore experiences such as: suffering, stigma, social attitudes, acceptance and coping. [13,68] Nor do we have adequate cross-cultural studies of these issues. Socio-cultural practices surrounding pregnancy and birthing practices can impede the implementation of training programs and other effective interventions that conflict with local practice. Psychological stress and social stigma remain barriers to participating in prevention interventions. A better understanding of the experience of preterm birth and stillbirth is essential to the successful and respectful implementation and scale-up of interventions.

#### Improving equity outcome measures in evaluation of interventions

Ethically sound intervention programs improve health outcomes as well as reduce health disparities within an intervention community, population, country or region. Despite substantial progress on the development of normative frameworks and practical strategies for priority-setting in health interventions, [50,52,66,69] there are very few evaluations of the effectiveness of interventions to prevent preterm and stillbirth that include assessments of equity as a clear outcome. At a very general level, there are three basic domains where decisions to scale-up interventions can incorporate considerations of equity:

1. *Equity of outreach*: implementing strategies to reach underserved segments of a population

2. *Equity of coverage*: achieving more comprehensive coverage with an intervention across all communities, with special attention to improving coverage among the poor

3. *Equity of impact*: demonstrating a decrease in the relative disease burden attributable to the intervention and outreach-coverage strategies among the poorest in the affected population [70]

While we have preliminary tools for measuring equity impact, many scientists either do not appreciate the importance of such measures or the additional analyses are not pursued because they often require increases in sample size, which can lead to higher study costs.

**Figure 2:**
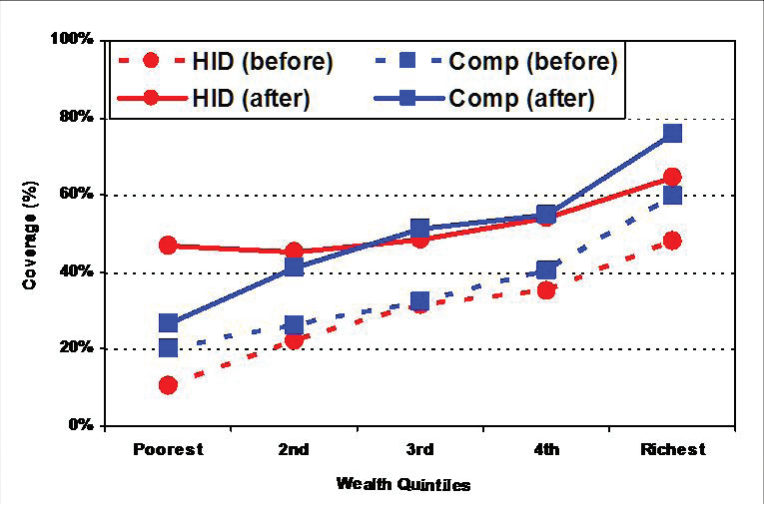
**Coverage with 3+ Antenatal Visits in HID (Intervention) and Comparison Districts.** Source: Bryce et al. 2008 [71].

A recent review of the UNICEF ACSD (Accelerated Child Survival and Development) in Mali showed that where both the intervention and comparison areas showed marked social gradients before the program was implemented in 2001, five years later access to antenatal care was significantly more equitable in districts with ACSD than in other districts. This strategy relied heavily on outreach sessions aimed at improving access to rural mothers living in remote areas, an example of equity in outreach [71]. This is one of very few examples where equity in population coverage was explicitly included in outcome measures, and equity of strategy discussed. Encouraging the inclusion of all three equity considerations for intervention reviews will help improve data on equity in maternal and child health, and will help inform discussions about improving both equity principles and outreach strategies with greater attention to different contexts (Figure 2).

In addition, it is important to consider equity measures over the long-term and with sensitivity to the "inverse equity hypothesis" associated with the introduction of new technologies [72], since evidence shows new technologies tend to be adopted by the wealthy and, therefore, are likely to increase health outcome inequities in the short-term.

#### Emerging issues in ethical decision-making and preterm birth

There are several emerging topics in parental decision-making, personal and social responsibility, and prevention of morbidity and mortality associated with preterm birth. The most significant gap is to distinguish the value decisions facing parents and providers in low- middle-, and high-income settings without conflating distinct cultural and socioeconomic contexts.

In HICs, successful delivery of interventions to prevent preterm birth and stillbirth will come up against social and cultural norms surrounding the freedom of choice in reproductive decision-making, particularly in the context of the United States. There has been some discussion of the ethical issues surrounding the use of assisted reproductive technology (ART) in high-income settings, including such issues as reducing multiples, resource allocation with expensive technologies, and parental choice [73,74]. Additional work needs to be done to address the attendant issues of: low birth weight and possible increases in morbidity associated with ART, inequity in access to ART as well as access to preterm birth support as a consequence of ART, guidelines for the responsible use of ART; and attitudes and beliefs surrounding the value trade-offs that parents must make surrounding the use of ART, such as delayed reproduction and increased risk for preterm birth. Emerging areas for social science research and the development of ethical guidance will explore women's choices and value tradeoffs in the workplace, delayed age of birth, occupational risks, and other decisions that may increase the likelihood of a preterm birth.

Discussion about the appropriate use of neonatal technology has dominated in high-income clinical settings. [75-81] However, there is very little understanding, beyond anecdotal accounts from those engaged in intervention efforts, about the range of moral issues that are pressing to women, parents and communities in LMICs. More country-specific social science data are needed that describe the context-specific and cultural issues facing women, parents and health providers across low- and middle-income settings, both rural and urban.

In low-income settings, for example, value questions surrounding birth spacing, distance from clinical facility, absence from work, or using scarce family resources to purchase antibiotics, are important value-trade-offs made everyday by mothers and parents living in rural areas often in poverty [82]. Having fewer high-tech options does not render value trade-offs regarding reproductive decisions any less important or any less difficult. Yet, there is almost no discussion of these challenges in the bioethics literature, and a copious discussion on value decisions in the use of NICUs, resuscitation, life-saving drugs, and other perinatal and neonatal interventions readily available in HICs. This is not to say that the ethical discussions surrounding the HIC dilemmas are unimportant, but rather, to highlight parallel and as yet invisible dilemmas occurring in low-income settings.

In middle-income settings, or transitional health systems, more data are needed to describe potential socioeconomic and cultural barriers to scale-up delivery of effective interventions to prevent preterm birth and stillbirth. Since out-of-pocket payments remain the primary means of financing health care—including childbirth in most of Africa and Asia [55,83,84]—attention is needed on the dilemmas facing mothers, parents and families in these regions, including special attention to gender issues in cultures that do not value girls [85]. Unlike most low-income communities, skilled health workers, access to facilities, and standard interventions and equipment are available in transitional economies. However, unlike most high-income countries, third party or state-sponsored payment of these services is not available. Value trade-offs pertaining to prenatal care and monitoring for high-risk pregnancies, C-sections, or decisions regarding life-saving interventions for preterm newborns will include difficult economic decisions for the parents and family, as well as the provider [86]. As in HICs, having better data on long-term morbidity of preterm survivors will be critical for parents and providers to make informed decisions during pregnancy and at the time of birth. However, better data on outcomes will not address the lack of health systems and economic support for families in LMICs aimed at preventing preterm birth and stillbirth, or at supporting preterm survivors.

In transitional socioeconomic regions there may also be resistance to accepting effective low-cost interventions, precisely because they are perceived to be low-cost and low-tech. Consider, for example, Kangaroo Mother Care (KMC), a very low-cost, low-tech intervention that encourages direct skin-to-skin contact between new-borns and mothers shown to support preterm survival (article 4 in this report [55]). An international survey of trainees in KMC from 25 LMICs reported several cultural and socioeconomic barriers to implementing this intervention, including the perception that KMC is the "poor man's alternative" to more sophisticated care. Trainees also reported objections to exclusive breastfeeding, given the perceptions among some that formula feeding is more modern and sophisticated [55,87]. There is as yet very little discussion of these dilemmas in the bioethics and social science literature. This represents an important area for future work.

There is a well-developed model for addressing ethical dilemmas that require balancing maternal autonomy in reproductive decisions with the interests of the developing fetus or infant [88]. The "balancing interests" framework has arisen in the context of caesarean deliveries, fetal surgery, brain death in pregnant women, maternal illness during pregnancy, intrauterine interventions, and infertility treatments. While offering valuable guidance in maternal-fetal conflicts and other decisions surrounding preterm delivery, much controversy remains over the best way to resolve maternal-fetal conflicts that involve situations due to circumstances out of one's control, such as being born into poverty. Known links between occupational and environmental stressors and preterm birth [89], for example, raise the previously discussed concerns about health disparities and preterm birth, and pose a challenge for maternal autonomy constrained by the tragic exigencies of poverty. The maternal-fetal interests model does not offer adequate guidance for such structurally determined choices surrounding pregnancy and warrants further discussion and research. The framework is also rooted in Western ethical traditions based on conflict and balance between opposing prima facie values or moral principles. There are many other rich ethical traditions that may cast these difficult choices surrounding preterm birth differently and can contribute to more culturally-appropriate ethical guidance on delivery of interventions. We need to encourage research and dialogue in this area to understand these variations.

#### Socioeconomic and cultural barriers to discontinuing ineffective interventions

Among the barriers to improving delivery of effective interventions to prevent preterm birth and stillbirth is how to discontinue practices and interventions that have been shown to be ineffective or harmful. This is a challenge in effective delivery in any area, but in the context of pregnancy it raises several ethical questions discussed in article 4 of this report [55].

Many entrenched but ineffective or harmful practices in rural and developing settings may be rooted in cultural practices surrounding childbirth. In middle- and high-income settings, overuse of ineffective or harmful interventions may reflect mothers' and families' demand for particular services, such as cesarean sections. Particularly in transitional health systems, such as in India, demand for such services may represent a broader or symbolic desire for status that comes with an increase in medicalized care [90]. Changing practices requires not only overcoming entrenched behavior but also the inertia and expense of established public health training programs. Retraining will also require sensitivity and engagement with existing cultural practices. In high-income or transitional economic settings discontinuing ineffective interventions will mean curbing the preferences and demand of women, parents and providers. In the case of elective cesareans attempts to curb overuse may be misinterpreted as placing unfair limits on women's reproductive choices. This case and others illustrate the critical importance of gathering and disseminating good data on ineffective interventions in a way that addresses social and cultural barriers to abandoning harmful or ineffective interventions.

## Conclusion

In the process of reviewing the scientific, medical, ethics, and social science literature on preterm birth and stillbirth a number of questions for research, normative analysis, public deliberation, or policy development have emerged (See Table 1). Ensuring these questions are at the top of the global health research, policy and bioethics agendas will contribute to a more thoughtful approach in our efforts to prevent global preterm birth and stillbirth. Clear bioethics research gaps remain along the research and delivery pathway. Addressing these gaps need not be an impediment to moving forward on the science, but rather has significant potential to support the effort. Particularly as attempts are made to consider diagonal funding and intervention programs—investment strategies in health systems development alongside targeted, or vertical, efforts to improve specific health outcomes—partnerships with social scientists and bioethicists can be especially valuable as the trade-offs required in competing programs represent significant value judgments. Bioethics has an opportunity to generate empirical data, shape public deliberation, and inform institutional changes and resource allocation decisions. This is needed to successfully deliver improved interventions for preventing preterm birth and stillbirth while addressing the deeper social, economic, and political issues that impact this important area in maternal, newborn and child health.

Considering preterm birth and stillbirth without borders with cross-cutting attention to HICs and LMICs, highlights important global differences in prevalence and causes but also identifies critical common ground. Globally, there is a common need to improve visibility of the complex and substantial disease burden associated with preterm births and stillbirths. There is a shared need to better understand the downstream impact on mothers, parents, preterm survivors, as well as communities, health systems, and educational systems. The challenge is exciting because it offers an opportunity to improve lives and address health disparities on several critical measures at once: neonatal survival, childhood morbidity and quality of life, women's health and quality of life, parental and family health. Identifying ethical common ground and encouraging public deliberation on areas of continued controversy can help shape a global research, development, and delivery agenda to prevent preterm birth and stillbirth.

The next and final article in this report presents an interdisciplinary action agenda to prevent preterm birth and stillbirth, and to improve related health outcomes globally [91].

## Authors' contributions

The article was written by MK. CR helped conceive of this article as part of a global report on preterm birth and stillbirth, and participated in its design, coordination, and review.

## Competing interests

The authors declare that they have no competing interests.
